# Functionalized Halloysite Nanotubes–Silica Hybrid for Enhanced Curing and Mechanical Properties of Elastomers

**DOI:** 10.3390/polym11050883

**Published:** 2019-05-14

**Authors:** Jing Lin, Dechao Hu, Yuanfang Luo, Bangchao Zhong, Yongjun Chen, Zhixin Jia, Demin Jia

**Affiliations:** Key Lab of Guangdong High Property and Functional Macromolecular Materials, School of Materials Science and Technology, South China University of Technology, 381 Wushan Road, Guangzhou 510640, China; 15521175271@163.com (J.L.); hdc1991@163.com (D.H.); pcbczhong@163.com (B.Z.); yjchen@scut.edu.cn (Y.C.); 18620901980m@sina.cn (Z.J.); psdmjia@scut.edu.cn (D.J.)

**Keywords:** rubber composites, nano hybrid, cure behaviour, mechanical properties

## Abstract

Vulcanization and reinforcement are critical factors in governing the ultimate practical applications of elastomer composites. Here we achieved a simultaneous improvement of curing and mechanical properties of elastomer composites by the incorporation of a functionalized halloysite nanotubes–silica hybrid (HS-s-M). Typically, HS-s-M was synthesized by 2-mercapto benzothiazole (M) immobilized on the surface of halloysite nanotubes–silica hybrid (HS). It was found that the HS-s-M uniformly dispersed in the styrene-butadiene rubber (SBR) matrix, offering more opportunity for M molecules to communicate with rubber. In addition, the physical loss of accelerator M from migration and volatilization was efficiently suspended. Therefore, SBR/HS-s-M composites showed a lower curing activation energy and a higher crosslinking density than SBR/HS composites. Moreover, a stronger interfacial interaction between HS-s-M and SBR was formed by the cross-linking reaction, giving a positive contribution to the eventual mechanical properties. The possible vulcanization and reinforcement mechanisms of SBR/HS-s-M composites were also analyzed in detail.

## 1. Introduction

Nano inorganic fillers have been established as the indispensable components for the reinforcement of most synthetic elastomers. In addition, the characteristics of nanofiller, such as structure, size, specific surface area and surface groups, play critical role in final mechanical properties and curing behaviour of elastomer composites [1,2,3,4]. Recently, to meet a variety of demanding performance requirements of elastomer products, nano hybrid fabricated by combining multi-nanoparticles with each other via the physical or chemical interaction has sparked the interests of scientists and engineers [5,6,7,8]. Kong et al. [9] prepared a highly hybridized nanofiller (CNTs/SiO_2_) by the self-assembly of silica nanoparticles and multi-walled carbon nanotubes, which could uniformly disperse in the rubber matrix; the corresponding composites exhibit high mechanical properties, excellent wet skid resistance and low rolling resistance. Jiang et al. [10] fabricated HNTs/CNTs hybrid through covalent bonding, achieving simultaneous reinforcement and toughening of polyurethane (PU) elastomers due to the synergetic effect of 3D structure of hybrid and hydrogen bonding interactions between hybrid and PU. Typically, in our previous work, a novel halloysite nanotubes-silica hybrid (HS) has been synthesized by the means of electrostatic self-assembly and in situ fabrication methods [11,12], which efficiently enlarge the specific surface area of HNTs through a unique three-dimensional nano-protrusions structure. It has been confirmed that this nano hybrid can achieve superior thermal stability and improved reinforcement in unsaturated polyester and SBR composites than pristine HNTs [12,13]. Nevertheless, the interfacial interaction between hydrophilic inorganic nano hybrid and hydrophobic elastomers is still weak, inevitably leading to the poor performance of elastomer composites.

On the other hand, the nano inorganic filler also has a significant influence on the vulcanization of elastomers [14,15]. It has been found that some rubber chains can be immobilized on the surface of nanofillers [16,17,18], and these immobilized rubber are difficult to be crosslinked due to the weak diffusibility of accelerators in the immobilized rubber layer [19]. In essence, the final performance and versatile practical applications of elastomers are strongly coupled with the curing state of both bulk rubber chains and immobilized rubber layer. Therefore, enhancing the vulcanization degree of the immobilized rubber chains may be an effective approach to further optimize mechanical properties of elastomer composites. Recently, the chemical anchor of vulcanization accelerator molecules on nanofillers surface offers an opportunity for improving the low vulcanization conversion of immobilized rubber layers [20]. This is because the anchored accelerators are exactly located in the immobilized rubber layers, increasing the collision chances between curatives. Besides, the organic accelerators can serve as the interfacial modifier of nanofillers to strengthen the interfacial interaction between nanofillers and rubber matrix. More importantly, the anchor of low molecular accelerator on nanofillers surface can simultaneously avoid the migration and volatilization at high temperature during rubber processing.

In this work, to achieve the simultaneous enhancement of curing and mechanical performances of elastomers composites, a functionalized nano hybrid (HS-s-M) was fabricated by 2-mercapto benzothiazole (accelerator M) chemically immobilized on the surface of HS via the linkage of silane coupling agent. The structure of HS-s-M was characterized by FTIR, XPS, TGA and TEM. The effect of HS and HS-s-M on the curing behaviour and mechanical properties of SBR composites were systematically investigated.

## 2. Materials and Methods

### 2.1. Materials

Halloysite nanotubes (HNTs) were supplied from Henan, China and purified according to the relevant method [21]. SBR (1502) was bought from the Sinopec company, Maoming, China, (3-chloropropyl) trimethoxysilane (CPTMS) came from Wanda Chemical Co., Ltd., Qufu, China, and Accelerator 2-mercapto benzothiazole (M), antioxidant N-isopropyl-N′-phenyl-1,4-phenylenediamine (4010NA), Ncyclohexyl- 2-benzothiazole sulfenamide (CZ) and Sulfur were industrial grade products supplied by Guangzhou Institute of Rubber Products, Guangzhou, China. Zinc oxide (ZnO), stearic acid (SA) and absolute ethanol were analytical reagents and used as is, without further purification.

### 2.2. Preparation of HS and HS-s-M

**Figure 1:**
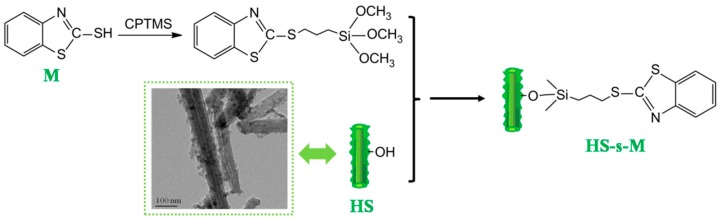
Schematic route of the preparation of HS-s-M.

HNTs-silica hybrid (HS) was synthesized according to our previous study [12]: 5.0 g of pristine HNTs was dispersed in 300 mL absolute ethanol and soniced for 0.5 h. Then, 6.0 g of TEOS was slowly dripped into the suspension. The mixture was stirred at 60 °C for 2 h to obtain the hybrid filler (HNTs-g-Silica). Finally, the solid was washed with ethanol for five times to remove the residual TEOS and dried in vacuum oven at 60 °C. The synthetic route of HS-s-M is illustrated in Figure 1. (3-chloropropyl) trimethoxysilane (CPTMS) (2g) was dispersed to a suspension of accelerator M (2 g) in absolute ethanol (150 mL) and stirred for 4h at 70 °C. Then, 10 g of HS was added into the above suspension to react for another 8h at 90 °C. The obtained sample was washed with absolute ethanol several times, the residues were dried in a vacuum oven at 70 °C, and the HS-s-M came out as a white solid.

### 2.3. Preparation of SBR/HS and SBR/HS-s-M composites

The composition of unfilled and filled SBR composites was summarized in Table 1. The total content of accelerator M including the part of HS-s-M was fixed 1 phr. SBR and rubber additives were compounded using a two-roll mill at room temperature. Then, the SBR compounds were press-cured for the optimum curing time (Tc_90_) at 160 °C.

### 2.4. Characterization of HS-s-M and SBR Composites

The structure of nano particles were characterized by Fourier transform infrared (FTIR) spectra and X-ray photoelectron spectroscopy (XPS, Kratos Analytical, Manchester, U.K.). FTIR was recorded on a Bruker Vector 70 FTIR spectrometer (Bruker Corporation, Billerica, MA, USA) ranging from 4000 to 400cm^−1^ with KBr pellets, and XPS was detected using a Kratos Axis Ultra with an aluminum Kα source. Thermogravimetric analysis (TGA) was employed to measure the weight loss of nano particles from 35 °C to 700 °C with NETZSCH TG209F1 (NETZSCH-Gerätebau GmbH, Selb, Germany) at a heating rate of 10 °C /min under nitrogen flow. Rubber compounds were cured by a UCAN UR-2030 moving-die rheometer (U-CAN DYNATEX Inc., Taiwan, China) at 160 °C.

The crosslinking density (*V_ε_*) of SBR composites was determined by means of equilibrium swelling method, with the following specific calculation formula [22]:

(1) $$V_{r}=\frac{m_{0}\times \phi \times \left(1-\alpha \right)/\rho_{r}}{m_{0}\times \phi \times \left(1-\alpha \right)/\rho_{r}+\left(m_{1}-m_{2}\right)/\rho_{s}}$$

(2) $$V_{e}=-\frac{\ln\left(1-V_{r}\right)+V_{r}+\chi {V_{r}}^{2}}{V_{s}\left({V_{r}}^{1/3}-V_{r}/2\right)}$$

where *V_r_* is volume fraction of swollen sample, *ϕ* is mass fraction of rubber, *α* is mass loss rate of sample during swollen process, and *ρ_r_* and *ρ_s_* are the density of vulcanized rubber and solvent, respectively. *V_s_* and *χ* are the molar volume of solvent and interaction parameters between rubber with solvent. The crosslinking density of SBR composites was obtained with the following procedure. Firstly, the measured sample (the weight was m_0_) was immersed in methylbenzene for three days at room temperature. After equilibrium swelling, the solvent of the sample surface was drained off with filter paper to determine the weight of residual sample (m_1_). Then, the obtained sample was dried on the vacuum oven at 70 °C until a constant weight was achieved, and the weight of the swollen sample was recorded m_2_. The dependence of the storage modulus on the strains of the unvulcanized and vulcanized SBR were determined on a rubber processing analyzer (Alpha, RPA 2000, Alpha Technologies, Hudson, Ohio, USA).

Heat capacity curves of the cuing reaction of SBR composites were achieved by a TA Q20 Differential Scanning Calorimetry (DSC, NETZSCH-Gerätebau GmbH, Selb, Germany) Instruments from 35 °C to 150 °C under oxygen flow at four heating rates (*β*), namely 3, 5, 10, and 15 °C/min. The apparent activation energy (*E_a_*) of the curing reaction of SBR composites were measured by Ozawa and Kissinger methods based on DSC as followed [23].

Ozawa method:

(3) $$Ea=-R\frac{\mathrm{dln}\beta }{d(1/Tp)}$$

Kissinger method:

(4) $$Ea=-R\frac{\mathrm{dln}(\beta /Tp^{2})}{d(1/Tp)}$$

where *T_p_* is the maximum peak temperature of DSC curve and *R* is the gas constant.

Heat capacity curves in the glass transition district (Tg) of SBR composites were obtained by a CA NETZSCH Instruments DSC 204 F1 from −80 °C to 20 °C at the heating rate of 20 °C/min under nitrogen atmosphere. Apart from this, the value of heat capacity step (Δ*C_pn_*) and the weight fraction of immobilized polymer layer (χ_im_) were fixed by the following equations [24,25]:

(5) $$\Delta C_{pn}=\Delta C_{p}/\left(1-\omega \right)$$

(6) $$\chi_{im}=\left(\Delta C_{p0}-\Delta C_{pn}\right)/\Delta C_{p0}$$

where Δ*C_p_* and Δ*C_pn_* refer to the heat capacity jump at the district of glass transition for polymer composites and unfilled polymer, respectively, and where *w* is the weight fraction of filler.

## 3. Results and Discussion

### 3.1. Characterization of HS-s-M

**Figure 2:**
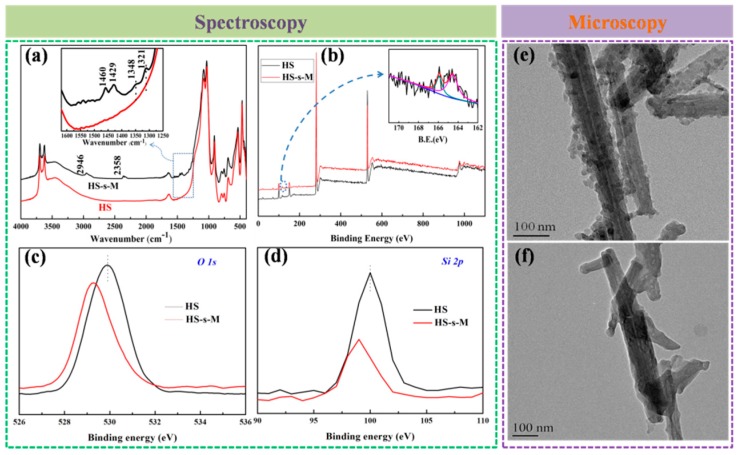
FTIR spectra (**a**) and XPS spectra (**b**) of HS and HS-s-M, High-resolution O 1s (**c**) and Si 2p (**d**) XPS spectra of HS and HS-s-M, TEM images of HS (**e**) and HS-s-M (**f**) (the insets in (**a**) and (**b**) are a higher magnification image of FTIR and high-resolution S 2p XPS spectra, respectively).

Hs-s-M was fully extracted with pure ethanol to remove the other residual chemicals before the structural characterization. The changes of functional groups and chemical bonds could be detected by FTIR and XPS. The FTIR spectra of HS and HS-s-M are depicted in Figure 2a. The noticeable characteristic band of HS-s-M at 2946 cm^−1^ ascribed to CH_2_ confirms the existence of a silane coupling agent. The peaks of 1461 and 1429 cm^−1^ in HS-s-M are attributed to the stretching vibration of C-H absorption band from benzene. The weak absorption at 1348 and 1321 cm^−1^ are assigned to C-N stretching vibration derived from accelerator M. However, no above visible characteristic bands are observed in HS. On the other hand, Figure 2b exhibits survey XPS spectra of the HS and HS-s-M. The double cleavage peaks near 165 eV in S 2p binding energy detected in the HS-s-M are attributed to two S elements of HS-s-M. To provide deep insights into the nature of bonding between HS and accelerator M, the high-resolution XPS corresponding to Si 2p and O 1s core binding energies were performed, as depicted in Figure 2c,d. Obviously, the binding energy of HS-s-M in O 1s is lower than that of HS. Besides, accelerator M immobilized on HS surface also induces a 1.02 eV decrease of binding energy in Si 2p. The red shifts of O 1s and Si 2p result from the the change of of electron densities, which further indicates accelerator M was chemically bonded on the HS surface. Comprehensively, the results of FITR and XPS indicate that accelerator M was successfully grafted on the surface of HS via the linkage of CPTMS.

The surface morphology of HS and HS-s-M can reflect their remarkable difference. As shown in Figure 2c,d, nano protrusions of silica are located on the surface of HNTs, while the surface of HS-s-M is not as rough as HS, due to the coating of organics on the surface of HS-s-M, further indicating the truth of successful preparation of HS-s-M.

**Figure 3:**
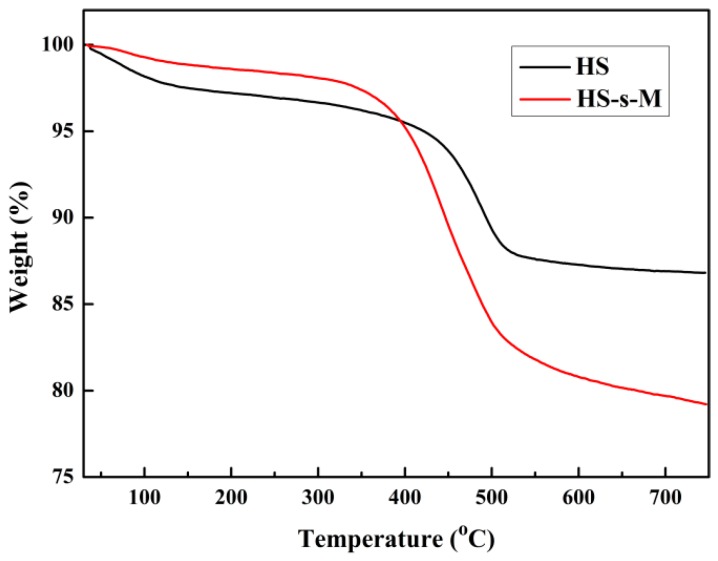
TGA curves of HS and HS-s-M.

A TG analysis of HS and HS-s-M were measured to quantitatively evaluate the contents of accelerator M on the surface of HS. As shown in Figure 3, HS gives a weight loss around 100 °C due to the physically adsorbed water on hydrophilic surface of HS. After the functionalization of organic accelerator M, the thermal stability of HS-s-M is decreased, which is assigned to the pyrolysis of silane coupling agent and accelerator M grafted on the HS-s-M surface. Based on the residual weight of HS and HS-s-M, the accelerator M content of HS-s-M was calculated as 2 wt.%.

### 3.2. Morphologies of SBR Composites Filled with HS and HS-s-M

**Figure 4:**
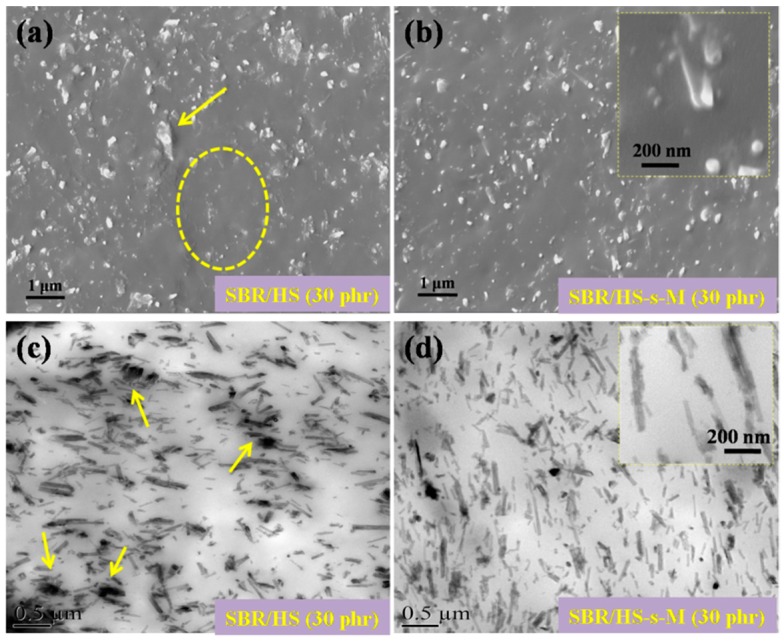
SEM images of SBR/HS (**a**) and SBR/HS-s-M (**b**) composites, TEM images of SBR/HS (**c**) and SBR/HS-s-M (**d**) composites (the insets in (**b**) and (**d**) are higher magnification images of SEM and TEM for SBR/HS-s-M composites, respectively).

Figure 4 illustrates the SEM (a, b) and TEM (c, d) images of SBR composites filled with the filler content of 30 phr. From Figure 4a, the SBR/HS composites exhibits large agglomerates of HS and “blank area” (no HS dispersed as emphasized by yellow circle in Figure 4a), indicating pristine HS is nonuniformly dispersed in the SBR matrix due to the polar hydroxyl groups on HS surface and high surface energy. These agglomerates and "blank areas" may act as stress concentration to deteriorate the mechanical properties of SBR composites under external stress [16]. As a contrast, after functionalization by organic accelerator M molecules, the HS-s-M is more homogenously dispersed throughout SBR matrix, as shown in Figure 4b. Moreover, it can be observed from the inset magnification in Figure 4b that HS-s-M is embedded in the SBR matrix with orientation, which profoundly influences the reinforcement of SBR composites. The TEM images in Figure 4c,d also present a similar dispersion state of pristine HS and HS-s-M in SBR matrix. Particularly, the vulcanization of elastomers is closely related to the dispersion of filler and curing agent in the elastomer matrix [26]. Therefore, the uniform-dispersed HS-s-M may effectively promote the vulcanization process of SBR composites apart from the outstanding reinforcement.

### 3.3. Curing Properties of SBR Compounds

**Figure 5:**
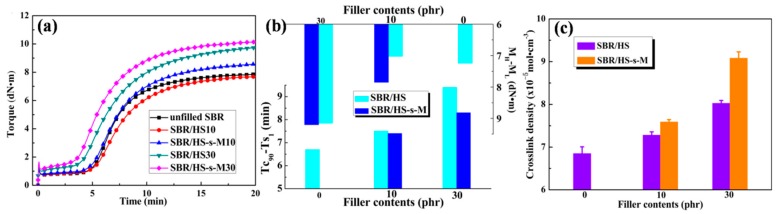
(**a**) Curing curves of SBR compounds at 160 °C, curing parameters (**b**) and crosslink density (**c**) of SBR compounds with different filler contents.

Figure 5a shows the curing curves of unfilled SBR and SBR compounds at 160 °C; corresponding curing parameters are summarized in Figure 5b. The curing curves of SBR compounds with the filler content of 10 phr nearly overlap with that of unfilled SBR compounds, demonstrating small amount of filler has little effect on rubber curing reaction. While the high filler content largely improves the torque of SBR compounds due to the enhanced filler-rubber interfacial interaction and filler–filler interaction [27]. Besides, the optimum cure time (Tc_90_) of SBR compounds increases with the filler content increasing. Considering the equal accelerator and sulphur, the addition of HS could impede the contact between rubber and additives, resulting in the decreased curing reaction rate of SBR compounds. The values of Tc_90_–Ts_1_ (the scorch time) and M_H_ (maximum torque)–M_L_ (minimum torque) usually refer to the production cycle and crosslinking density of rubber compounds. As shown in Figure 5b, Tc_90_–Ts_1_ of SBR/HS-s-M compounds is lower than that of SBR/HS compounds, while the M_H_–M_L_ of SBR/HS-s-M compounds is higher, which implies HS-s-M could efficiently reduce the production cycle and improve the crosslinking density of SBR composites. The crosslink density of SBR composites shown in Figure 5c exhibits a consistent trend with the results of M_H_ –M_L_. That could be attributed to that the higher use efficiency of accelerator and stronger interfacial interaction between HS-s-M and rubber matrix.

### 3.4. Curing Kinetics of SBR Compounds

**Figure 6:**
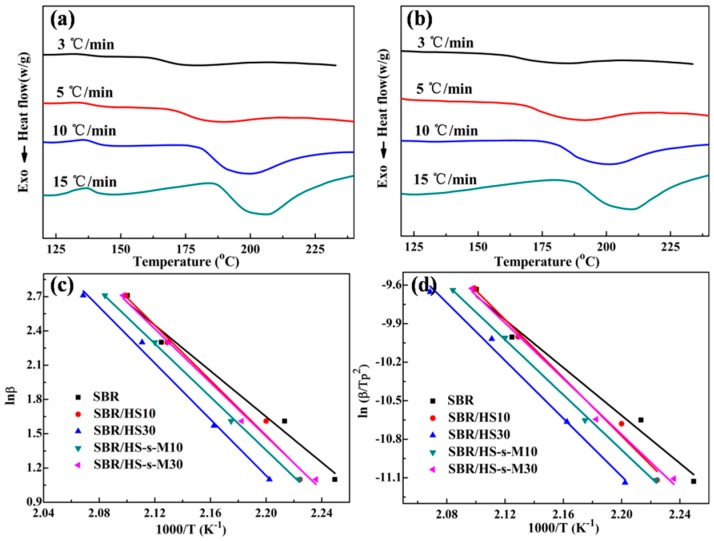
Typical dynamic DSC curves of (**a**) SBR/HS30 compounds and (**b**) SBR/HS-s-M30 compounds; linear fitting for calculating apparent activation energy (Ea) by the (**c**) Ozawa method and (**d**) Kissinger method.

DSC was introduced to investigate the kinetics of curing reaction of SBR compounds. The typical dynamic DSC curves of SBR compounds filled with 30 phr filler are exhibited in Figure 6 (the DSC curves of SBR and SBR compounds with the filler of 10 phr are not shown here), the specific curing parameters are illustrated in Table 2. The initial curing temperature (*T_onset_*) and the peak temperature (*T_p_*) of SBR compounds have a trend of increment as the heating rate increases. It is more readily acknowledged that the diffusion of rubber chains and additives are sluggish because the temperature response cannot keep up with the variation of heating rate, leading to the postponement of rubber vulcanization. Furthermore, *T_onset_* and *T_p_* of SBR/HS composites increase remarkably with the increasing filler at certain heating rate due to the fact that filler hinders accelerator molecule to communicate with rubber, and hence slows down the curing reaction rate of rubber, which is consistent with the results of curing curves. However, it is interesting that *T_onset_* and *T_p_* of SBR/HS-s-M compounds have no significant changes with increasing filler, indicating the functionalization of hybrid filler could compensate the slow curing reaction induced by the filler hindering. Meanwhile, the enthalpy value (Δ*H*r) during the rubber vulcanization of SBR/HS-s-M compounds is higher than that of SBR/HS compounds. Take SBR compounds filled with 10 phr filler for example: Δ*H*r of SBR/HS-s-M compounds is 15.96 J/g compared to 12.83 J/g for SBR/HS compounds when the heating rate is fixed 3 °C/min. Higher Δ*H*r of SBR/HS-s-M compounds suggests that HS-s-M could accelerate rubber vulcanization and improve the crosslinking densitiy of rubber composites [28]. The curing activation energy (*Ea*) of SBR compounds calculated according to the slope of linear fitting (as shown in Figure 6c,d) were summarized in Table 2. Obviously, *Ea* of unfilled SBR is lower than that of filled SBR compounds, owing to the volume effect of filler [29,30]. However, in the filled SBR compounds, the curing agents are hindered by the filler and have to diffuse through tortuous paths to participate the vulcanization process, leading to high activation energy. Fascinatingly, SBR/HS-s-M compounds present lower *Ea* by contrast to that of SBR/HS compounds, revealing that immobilization of accelerator on the hybrid surface could significantly reduce the corresponding energy barrier and accelerate cross-linking reaction.

### 3.5. Proposed Vulcanization Mechanisms of SBR Compounds

**Figure 7:**
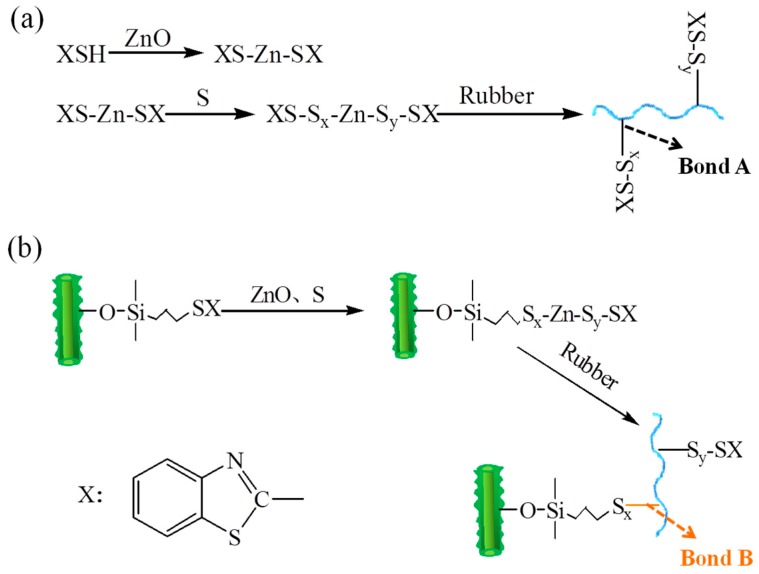
The possible vulcanization mechanisms of SBR compounds.

According to the vulcanization mechanisms of rubber with the benzothiazole accelerated sulphur system proposed by some researchers [31,32], the possible vulcanization reactions of SBR filled with immobilized accelerator are depicted in Figure 7a,b. In the initial stage of curing, 2-mercapto benzothiazole is transformed into Zn^+^ coordinated complex via a series of reactions in the presence of ZnO and S. This complex is highly unstable, eventually leading to the crosslink of rubber via polysulfur bonds, namely “Bond A”, as shown in Figure 7a. In SBR/HS-s-M compounds, a free accelerator anticipates in the same curing reaction just as for SBR/HS compounds. Simultaneously, a Zn^+^ coordinated complex included filler and an accelerator generate a new reaction between the rubber and the hybrid filler via crosslink bond of “Bond B” (in Figure 7b). Diverse chemical crosslink bonds in SBR/HS-s-M compounds contribute to the higher crosslink density than that of SBR/HS composites, even when they are filled with the equal accelerator. Moreover, the accelerator molecules of HS-s-M are located exactly in the interfacial region where the immobilized rubber is located, increasing the collision chances of curatives in the immobilized rubber layers. On the other hand, a low molecular accelerator M is easily volatile and migratory, which would inevitably reduce the use efficiency of sulphur and the vulcanization rate. Conversely, the HS-s-M is uniformly dispersed in the rubber matrix and not easily migratory. Therefore, based on the above discussion, the denser crosslink network together with lower physical loss of curatives should be responsible for the higher crosslink density and lower *Ea* of SBR/HS-s-M compounds than that of SBR/HS compounds. Predictably, a stronger interfacial interaction between SBR and HS-s-M by the linkage of "Bond B" will give a positive contribution to the mechanical properties of SBR/HS-s-M composites.

### 3.6. Interfacial Interaction between SBR Matrix and Filler

**Figure 8:**
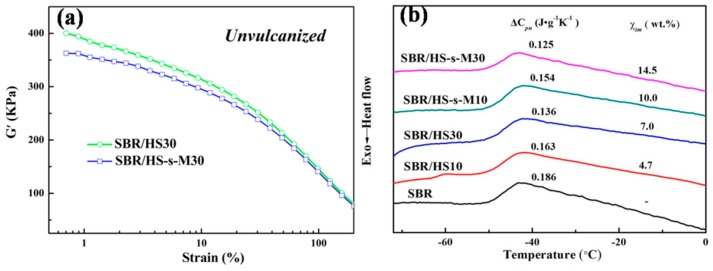
Dependence of the storage modulus (G’) of unvulcanized SBR compounds with 30 phr filler on strains (**a**), DSC curves and weight fraction of immobilized rubber layer (χ*_im_*) of SBR composites (**b**).

The interfacial interaction between the SBR matrix and the filler has a significant influence on the final mechanical properties. Figure 8a depicts the dependence of the storage modulus of unvulcanized SBR compounds filled with 30 phr HS and HS-s-M. It was found that SBR/HS compounds presents higher G’ than SBR/HS-s-M, indicating that the unfunctionalized HS can form some aggregation due to the hydrogen-bond interactions between unfunctionalized HS. In addition, the lower value of *G’* for SBR/HS-s-M compounds is ascribed to the improvement of compatibility between rubber and modified HS, consistent with the morphological observation presented above. Moreover, as shown in Figure 8b, the weight fraction of immobilized polymer layer (χ*_im_*) approaching filler was investigated by DSC. The χ*_im_* can be defined as the relaxation degree of rubber affected by filler. Therefore, high χ*_im_* demonstrates more rubber chains are bonded on filler surface, due to strong interfacial interaction between filler and matrix. Clearly, at the same content of filler, χ*_im_* is in order of value as followed: χ*_im_* (SBR/HS-s-M) > χ*_im_* (SBR/HS), which indicates a stronger interfacial interaction between SBR and HS-s-M induces more rubber chains are immobilized on filler surface. DSC results are in good accordance with RPA, providing hard evidence for the positive mechanical properties below.

### 3.7. Mechanical Properties and Reinforcing Mechanism of SBR Composites

**Figure 9:**
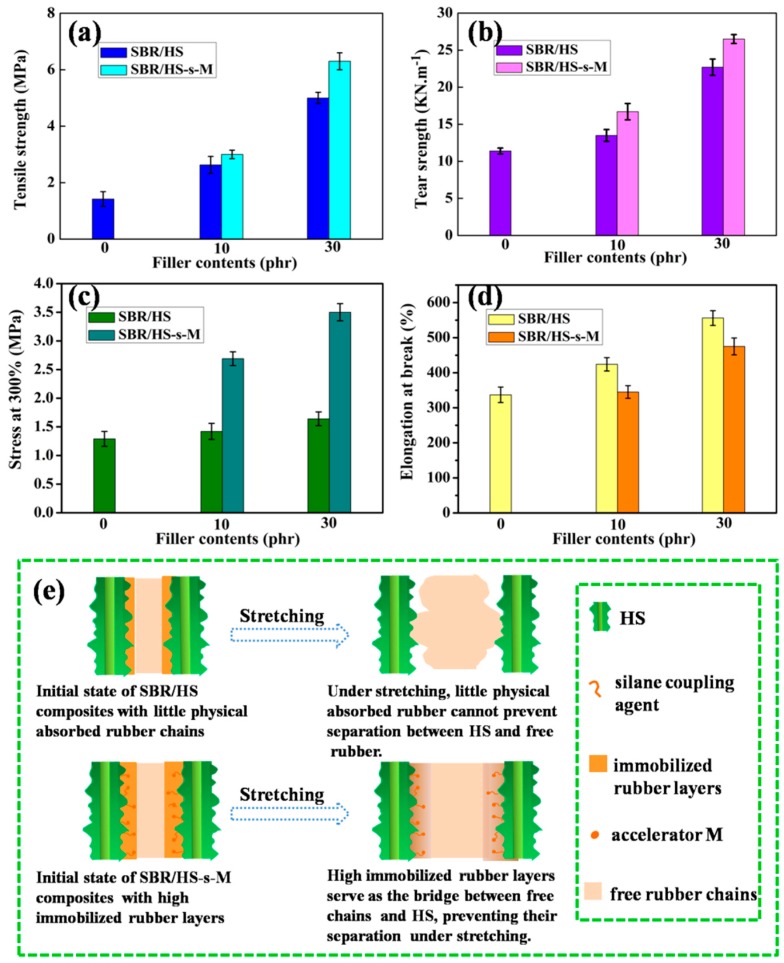
(**a**) Tensile strength, (**b**) Tear strength, (**c**) Stress at 300%, and (**d**) Elongation at break of SBR composites; (**e**) proposed model for the structural change of rubber chains in the rubber-filler interfacial region during stretching.

The mechanical properties of unfilled SBR and SBR composites are comparably evaluated in Figure 9, and the corresponding reinforcing mechanism is also analyzed. From Figure 9a–c, it can be observed that the tensile strength, tear strength and modulus (always estimated by the stress at 300%) of SBR composites are all improved by the incorporation of fillers. Especially, the SBR/HS-s-M composites give higher values than the composites with pristine HS. However, as shown in Figure 9d, the SBR/HS-s-M composites present a lower elongation at break than SBR/HS composites. This is because the slippage of SBR chains on HS-s-M surface during stretching is effectively restricted, due to the elevated interfacial interaction between HS-s-M and the rubber matrix. To better understand the structural change of rubber chains in the rubber-filler interfacial region under stretching, the proposed model is depicted in Figure 9e. Due to the functionalization of silicone coupling agent and accelerator, thicker immobilized rubber layers are formed on HS-s-M surface than pristine HS, which can serve as the bridge between filler and free rubber chains. Under equal stretching conditions, more rubber chains in SBR/HS-s-M composites are orientated between the neighboring HS-s-M. The stress loaded on SBR/HS-s-M composites can be effectively transferred from bulk rubber to HS-s-M by the stretched straight rubber chains and more energy is dissipated. On the contrary, the little physical absorbed rubber chains approaching hydroxyl HS are difficult to form effective chain orientation between HS and free rubber [33]. It is known that the rubber chain orientation is directly related to the strength of rubber composites [34]. Consequently, SBR/HS-s-M composites exhibit higher mechanical performances than SBR/HS composites.

## 4. Conclusions

A type of functionalized halloysite nanotubes–silica hybrid, HS-s-M, was successfully fabricated in this work. It was found that HS-s-M significantly reduced the irreversible agglomerates of HS in SBR matrix and achieved strong interfacial interaction with SBR. Besides, the physical loss of low molecular accelerator M was efficiently suspended. As a result, SBR/HS-s-M composites showed the lower curing activation energy and higher crosslinking density than SBR/HS composites even containing equivalent accelerator M. Moreover, the ultimate mechanical properties of SBR/HS-s-M composites were largely enhanced compared to SBR composites with pristine HS. In conclusion, this work achieved synergistic enhancement of curing and mechanical properties of elastomer composites by the functionalized nanohybrid, which may give a valuable inspiration for the preparation of high performance rubber composites.

## Figures and Tables

**Table 1:** Formulations of SBR compounds.

Samples	SBR	HS	HS-s-M	CZ	M	4010NA	SA	ZnO	S
SBR	100	-	-	1	1	2	2	5	1.6
SBR/HS10	100	10	-	1	1	2	2	5	1.6
SBR/HS30	100	30	-	1	1	2	2	5	1.6
SBR/HS-s-M10	100	-	10	1	0.8	2	2	5	1.6
SBR/HS-s-M30	100	-	30	1	0.4	2	2	5	1.6

**Table 2:** Vulcanization characteristics and apparent activation energy (Ea) of SBR compounds obtained from DSC.

Samples	β (°C/min)	T_onset_ (°C)	T_peak_ (°C)	ΔHr (J/g)	Ea (kJ/mol)	
					Ozawa	Kissinger
SBR	3	157.74	171.55	13.31	83.2	77.4
	5	164.76	178.81	17.08		
	10	176.01	197.65	16.17		
	15	184.8	203.12	13.57		
SBR/HS10	3	158.2	176.56	12.83	100.3	92.7
	5	166.01	181.55	13.83		
	10	180.22	196.72	16.65		
	15	186.97	203.4	16.03		
SBR/HS30	3	160.9	181.05	10.35	101.4	93.6
	5	168.4	189.43	11.71		
	10	180.39	200.74	11.81		
	15	187.44	210.43	10.31		
SBR/HS-s-M10	3	161.65	174.25	15.96	96.8	89.2
	5	170.3	185.19	13.56		
	10	181.53	196.39	16.6		
	15	187.84	203.82	16.1		
SBR/HS-s-M30	3	161.94	176.66	10.86	97.7	90.1
	5	170.44	186.79	12.76		
	10	180.7	198.7	13.91		
	15	187.32	206.8	10.97		
